# A co-design living labs philosophy of practice for end-to-end research design to translation with people with lived-experience of mental ill-health and carer/family and kinship groups

**DOI:** 10.3389/fpubh.2023.1206620

**Published:** 2023-11-30

**Authors:** Victoria J. Palmer, Jennifer Bibb, Matthew Lewis, Konstancja Densley, Roxanne Kritharidis, Elise Dettmann, Pam Sheehan, Ann Daniell, Bev Harding, Tricia Schipp, Nargis Dost, Gregor McDonald

**Affiliations:** ^1^Primary Care Mental Health Research Program, The Department of General Practice and Primary Care, Melbourne Medical School, Faculty of Medicine, Dentistry and Health Sciences, The University of Melbourne, Parkville, VIC, Australia; ^2^The ALIVE National Centre for Mental Health Research Translation, The University of Melbourne, Parkville, VIC, Australia; ^3^Co-Design Living Labs Program Members, Primary Care Mental Health Research Program, The Department of General Practice and Primary Care, Melbourne Medical School, Faculty of Medicine, Dentistry and Health Sciences, The University of Melbourne, Parkville, VIC, Australia

**Keywords:** experience co-design, co-design, living labs, lived-experience, mental health, research design, implementation, mental health research translation

## Abstract

There is increased recognition that people with lived-experience of mental ill-health ought to be centred in research design, implementation and translation, and quality improvement and program evaluation of services. There is also an increased focus on ways to ensure that co-design processes can be led by people with lived-experience of mental ill-health. Despite this, there remains limited explanation of the physical, social, human, and economic infrastructure needed to create and sustain such models in research and service settings. This is particularly pertinent for all health service sectors (across mental and physical health and social services) but more so across tertiary education settings where research generation occurs for implementation and translation activities with policy and services. The Co-Design Living Labs program was established in 2017 as an example of a community-based embedded approach to bring people living with trauma and mental ill-health and carers/family and kinship group members together with university-based researchers to drive end-to-end research design to translation in mental healthcare and research sectors. The program’s current membership is near to 2000 people. This study traces the evolution of the program in the context of the living labs tradition of open innovation. It overviews the philosophy of practice for working with people with lived-experience and carer/family and kinship group members—togetherness by design. Togetherness by design centres on an ethical relation of *being-for* that moves beyond unethical and transactional approaches of *being-aside* and *being-with*, as articulated by sociologist Zygmunt Bauman. The retrospective outlines how an initial researcher-driven model can evolve and transform to become one where people with lived-experience of mental ill-health and carer/family kinship group members hold clear decision-making roles, share in power to enact change, and move into co-researcher roles within research teams. Eight mechanisms are presented in the context of an explanatory theoretical model of change for co-design and coproduction, which are used to frame research co-design activities and provide space for continuous learning and evolution of the Co-Design Living Labs program.

## 1 Introduction

Embedding lived-experience (or what is also termed within the published literature as service users, experts-by-experience, and within government reports/policies as consumers and/or carers perspectives) within research, service design/re-design and systems re-design, and healthcare improvement has evolved into a wider trend of participation called the Participatory Zeitgeist, or “the spirit of our times” (1). This Zeitgeist has been driven by a confluence of social, cultural, political, and economic forces that permeates all sectors and indeed much of our public and personal lives (2). This spirit of our times has led to a dramatic increase in ‘co’ practices and recognition from social, academic, and political circles of the importance of experiential knowledge as evidence-based approaches. However, the extent to which this experiential knowledge is afforded equivalent weighting within the established hierarchy of evidence applied in research, policy, and service design and practice is limited (3–5).

Additionally, the growth in “co” practices has led to an increase in what has been termed co-biquity (6). This has been defined as “an apparent appetite for participatory research practice and increased emphasis on partnership working, in combination with the related emergence of a plethora, of ‘co’ words” (6). Although part of the co-biquity challenge is that many of the participatory methods and practices outlined as co-design or coproduction and other collaborative terms are rarely evaluated against core criterion such as who has been involved, how have people been engaged in collaborative processes of designing together, and what was designed or made for change or implemented as a result. Similarly, it is rare to see an evaluation of the extent to which co-design processes, methods, and outcomes have addressed structural and interpersonal inequalities in power and decision-making (7). Where evaluation material is available, it is largely qualitative interview reports of people’s experiences participating in co-design projects for service improvement (8).

Over a decade ago, authors in co-design fields began to raise concerns over the dilution and conflation of meanings and practices from collaborative traditions and the misappropriation of the terms co-design and coproduction (9). Such broad usage of coproduction and co-design terminology across a range of research disciplines (for example, urban planning, public management, environmental studies, design fields broadly, and education) and within healthcare quality improvement and in service design/re-design, has seen participatory approaches adopted in expansive ways. The pendulum has swung further in the co-design field when looking at healthcare quality improvement practices and what might be termed ‘mainstream’ service design/re-design processes. Where co-design once was defined as “the creativity of designers and people not trained in design working together in the design development process” (10), it has now come to focus on a central role for people with lived-experience (9, 11).

Historically, published literature has been replete with reference to the concept of “user/s” to define the goal for design to centre user’s needs and perspectives (12). This phenomenon of user participation is not all that new; participatory design definitions and practices have been premised on this also (11). Lucero and Vaajakallio (13) reported that “researchers have started to see ‘everyday people’ not only as the recipients of the artefacts of the design process, but as active participants in the design and production process itself, capable of adapting products to better meet their own needs”. In short, as Steen has argued previously, co-design thus reflects “an instance of moral [e]nquiry…” and a return to pragmatist ethics where the “return to ordinary life-experiences of inherently social, embodied, and historically situated beings” (9) is key.

In the current context of co-design, however, lived-experience-centred models mean more than a workshop with users about the appropriateness or usability of a product or technology, more than the ordinary and situated life experience, and more than user engagement. Quality and service improvement fields quite rightly are about bringing “service providers, service users and other relevant stakeholders [together to] use design tools and methods to work collaboratively to ensure service provision is informed by their shared experiences” (14). In mental healthcare (both in relation to research and to the delivery of care), significant power disparities exist, and indeed human rights abuses have occurred. Thus, active participation through methods that centre experience is essential to ensure that the goals of social justice are met. In this respect, experience-based processes of co-design are core to working with existing inequities and human rights abuses and exploring experiential injustices. The current emphasis on mental health reform, at least in Australia, also means that it is critical to consider how people with lived-experience may increasingly lead or co-lead co-design processes (15). That means there is a need to attend to co-analysis and interpretation of the results of co-design so that epistemic injustices (how knowledge is formed, shared, implemented, and evaluated) are not repeated inadvertently (5). In the shorter term, where co-design is espoused, there is a need for actively attending to how power was re-balanced and where designers were positioned within co-design processes. We need to shed light not only on what happened in co-design but also on what was implemented, where it might have led to change, and what the impacts of this might have been.

While mainstream service design/re-design, quality improvement, and systems transformation efforts have grappled with the implementation of co-design for some time now, there has been less examination of how to configure academic, university, and other research settings to embed lived-experience-centred models for research implementation and translation efforts (4, 12). Given the hierarchical nature of academic contexts and the diversity of mental health research disciplines, this is challenging, and there is a need to evaluate how lived-experience is being somewhat uncomfortably positioned as an indicator of political and social recognition of inclusion (16, 17). Centring lived-experience is particularly important in spaces where people have experienced systemic injustices and possibly significant harm and have not had the full protection of human rights and recognition of their voices. In these circumstances, people may not have been included in decisions about service design, development, or what programs are offered and how care is delivered. Inclusion alone, however, is not an indicator of political and social recognition. Increasingly, literature is emerging on co-designed interventions or co-design for research projects, and it can be an expectation by funders to illustrate co-design (or consumer and community participation as government agencies word it) in research grants (18). This gives cause to consider the kind of research architecture that is needed to embed lived-experience within end-to-end research design to translation using co-design. It also means we must pay close attention to how lived-experience is understood and included in co-design efforts.

Contemporarily, lived-experience refers to both working collectively with people who have direct experiences of the topic, issues, or problem in focus for co-design and the interaction of experiential knowledge within co-design, ensuring epistemic justice (valuing of experiential knowledge) is achieved (5). It includes attention to the framings for co-design, being clear about what social justice issues are being addressed, and how co-design is explicitly addressing power imbalances (19). In more recent developments in mental health research, suicide prevention, and within First Nations methodologies and Aboriginal and Torres Strait Islander social and emotional wellbeing programs, lived-experience has become key to addressing harms that have been experienced with the removal of rights and human rights abuses. Defining the parameters of lived-experience within co-design has become important, and ensuring lived-experience reflects “direct, first-hand substantive experience of mental distress, illness, diagnosis and/or mental health services. [Or] as associated with Lived Experience of poverty, trauma and other forms of prejudice and discrimination (e.g., racism and ableism)” (20) (p. 3) is fundamental. Publications on the importance of inclusion and lived-experience leadership expand upon substantive experience to suggest that diverse qualities are held and enacted by people with lived-experience, which generate change within and across mental health and social sectors (such as championing justice, centring lived-experience, and building relationships with peers and allies) (17). In the context of Aboriginal and Torres Strait Islander communities, lived-experience is expanded to “recognise the effects of ongoing negative historical impacts of colonisation or specific events on the social and emotional wellbeing of Aboriginal and Torres Strait Islander peoples. It encompasses the cultural, spiritual, physical, emotional, and mental wellbeing of the individual, family, or community” (21). Attention to lived-experience has become key to ensuring social justice and issues of inequity, and structural inequalities are in focus within co-design.

In the Co-Design Living Labs program and its philosophy of practice, lived-experience is described and applied as “community-led lived-experience.” This means that people engage as members of the program (referred to in our current day-to-day activities and engagements as co-designers) with their direct, personal experiences of mental ill-health and service systems or support expertise as carer/family and kinship groups. Importantly, there may also be nuances and elements of lived-experience located from identities in community stories, events, and happenings that are critical to the framing and shaping of experience. Therefore, conveners of co-design aim to always work closely with how the communities we are collaborating with shape and define lived-experience from the perspectives and positionalities of people within co-design. Community-led lived-experience thus acknowledges a need to recognise framings that may encompass cultural, social, and political differences to direct experience. This includes attention to the appropriateness of methods that are adopted within co-design.

This study outlines the establishment and evolution of the Co-Design Living Labs program and its philosophy of practice within The University of Melbourne between 2017 to present. It documents the establishment and transitions of the program from researcher-led to increasingly co-designer-led over this period. By philosophy of practice, we refer to the values upheld in our study, the roles and responsibilities of our ways of working together for change in implementation and translational research, and the component parts required for the operationalisation of the program. We call this philosophy of practice “togetherness by design.” The philosophy of practice couples theoretical work from sociologist Zygmunt Bauman’s articulation of three forms of togetherness: being-aside, being-with, and being-for (22). Co-design is understood as the “co” equating with togetherness and therefore the practices being about “designing together”. However, in keeping with co-design traditions, designing together means thinking about both what is made and how that making attends to social justice. It includes an evaluation of supported and shared decision-making processes that were applied and how power imbalances were addressed as they play out in the living labs’ tradition of open innovation and collective empowerment (23).

## 2 Materials and method

The Co-Design Living Labs program was founded within the Primary Care Mental Health research group in 2017. The program is now expanding as part of a national network in the ALIVE National Centre for Mental Health Research Translation funded by the Australian National Health and Medical Research Council 2021–2026 (GNT2002047) as part of a Special Initiative in Mental Health. The ALIVE National Centre’s mission is transforming mental health and wellbeing through primary care and community action. Its vision is for vibrant communities that support mental health and wellbeing to enable people to thrive. There are currently 17 university partners engaged in the Centre’s work, with membership growing across three networks supporting research program implementation and translation. The Centre grows lived-experience research capabilities within a tailored arm of a Next Generation Researcher network called the Lived-Experience Research Collective. An Implementation and Translation network is focused on growing capabilities and a national infrastructure to support adaptive co-design, demonstration projects, and promising models. The Co-Design Living Labs network will connect co-design programs across universities to expand end-to-end research design to translation. This builds on the aim of the Co-Design Living Labs program to create a purposeful space for people with lived-experience of mental ill-health and carers/family and kinship group members to co-create research and translation activities. Since its establishment in 2017, the program has grown from a membership of 600+ people to current membership of nearly 2000 people across Victoria and other states and territories of Australia. In this retrospective, we mark the transitions from an initially researcher-driven operational model set up by the lead author, who has lived-experience of mental ill-health (24), to one where co-designers now identify priorities for research and where their perspectives are shaping the research questions and approaches that are developed. It is important to acknowledge that the personal experiences of the lead author in navigating the re-definition of self that comes with lived-experience was a key motivator for program establishment. This included a view that there was a need to improve community-led mental health research and for better engagement processes in university-based research (25). These foundational values mean that lived-experience has shaped the co-design practices and processes undertaken since inception. More recent transitions in the program now include that co-designers have moved to co-researcher roles (which we will explain later) and an Aboriginal and Torres Strait Islander co-design research lead has been appointed in 2021. This study does not outline the transition to the inclusion of Aboriginal and Torres Strait Islander-led work; this will be detailed in a separate article illustrating the role of Indigenous Knowledge Systems whereby co-design may be articulated with different cultural practices (26). The future goal is that co-designers in the current program will adopt the living labs as a social enterprise utilising cooperative, democratic structures to maintain a commitment to the important issues of justice, power, and shared decision-making. This would support co-designers to drive the research agendas and co-convene the activities of the program with direct fiscal benefit flowing to them.

### 2.1 Recruitment to the co-design living labs program

The Co-Design Living Labs program membership base was grown by inviting former research participants (people with lived-experience) from completed mental health research studies to join. Two longitudinal studies were completed in the primary care mental health research program in 2016 and 2017: (a) a world-first stepped wedge cluster randomised trial (9) of an adapted mental health experience co-design approach for service improvement and psychosocial recovery outcomes, with 287 people living with conditions described in the literature as severe mental illness (herein referred to as mental ill-health)—the CORE Study (2012–2017) and (b) the diamond study (17) exploring over 700+ people’s experiences of living with depression and health services use (2003–2016). Completion of these flagship studies provided a turning point and an opportunity to shift away from what may be characterised as transactional research processes and agendas to relationally oriented practices.

#### 2.1.1 The adoption of a living labs approach

The living labs concept was identified as an open innovation pathway for mental health research to build on cooperative traditions and relational practices. It was adopted with a view to a future cooperative structured social enterprise being established from the program, as mentioned in the above section. Additionally, we sought to disrupt the idea that research practices located within a medical setting are only about scientific lab-based research, which has historically been characterised as having limited engagement of the public. Living labs in these environments have also tended to foster a test bed model where users are the objects of study rather than co-creators (12). Here, the term living lab was adopted to signal life, living, being alive and dynamic, and the importance of working with people in medical and health research activities every day with lived-experience. Bringing co-design and living labs together enabled the program to foreground social justice, power, and shared decision-making in activities.

In the literature, four key traits of living labs have been described: (1) a purpose to innovate products and services; (2) co-creation with users; (3) completion of activities in real-life settings; and (4) fostering public–private partnerships (27). These traits are essential where the focus is on innovation and co-creation, and they enable our program and future network-based activities to effectively operate an *anywhere, everywhere living laboratory* focused on real-life settings. This fits with the European Network of Living Labs (ENoLL) definition of living labs as “user-centred open innovation ecosystems based on a systematic user co-creation approach, integrating research and innovation processes in real-life communities and settings” (28). Our approach, however, expands the living labs’ tradition from a grounding in social innovation, partnerships, and open approaches to operationalisation of these factors within a lived-experience context. Therefore, we intentionally brought together the practices of Co-Design with Living Labs for the naming and setup of the program (19, 29).

**Figure 1:**
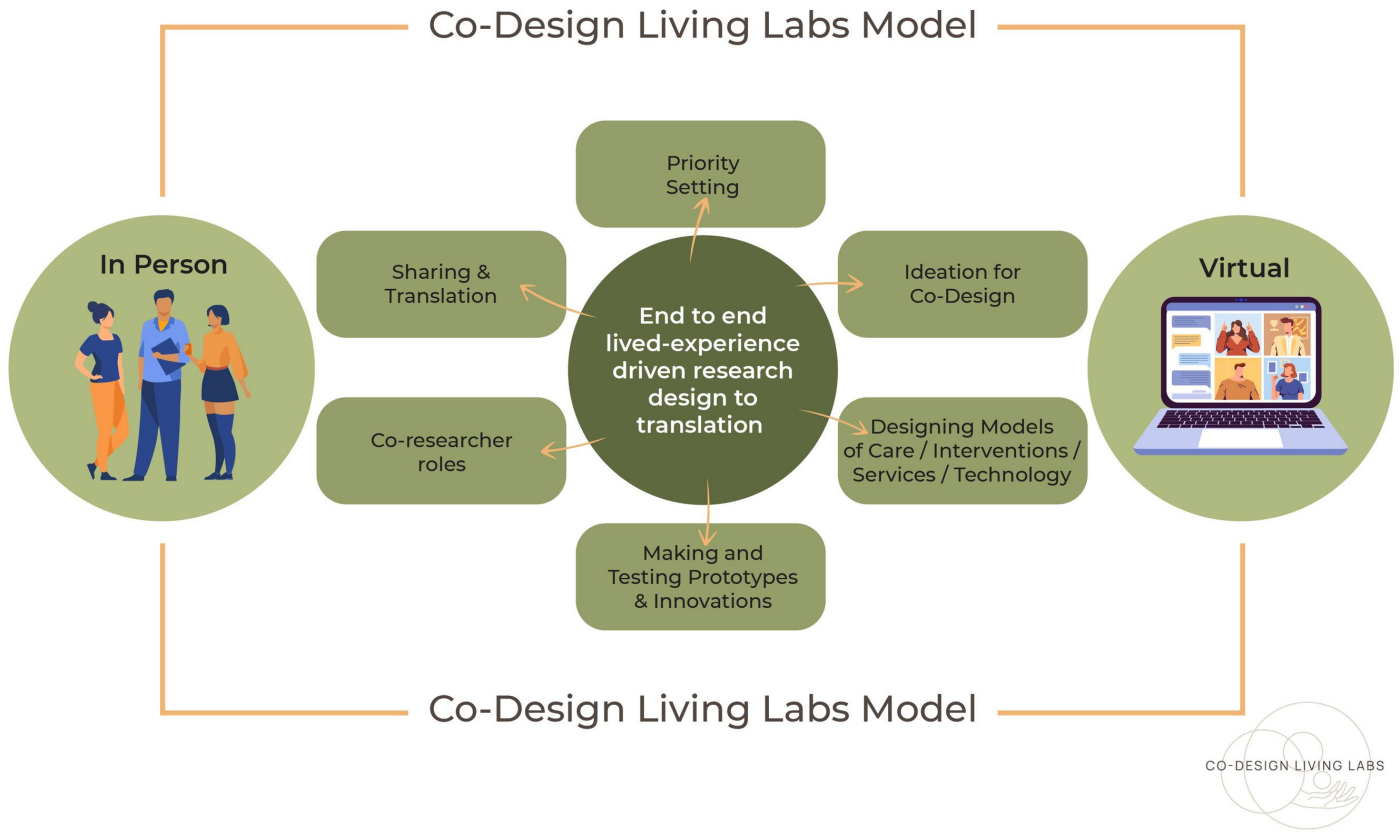
The co-design living labs operating model.

In this relational form of engagement, the goal was to ensure lived-experience became embedded within the priority setting of research grant proposals and the establishment of research questions. This included the ideation on component parts of these grants or other innovations, the co-creation of new models of care (also termed interventions in the health sciences literature) and healthcare technologies and processes, and the making and shaping of prototypes (using paper to technology-facilitated approaches, and with co-research and dissemination and communication). These aforementioned goals form the foundations of the Co-Design Living Labs operating model, which is presented in Figure 1.

In the next section, we provide an overview of the evolution of the Co-Design Living Labs philosophy of practice called ‘togetherness by design.’ This philosophy of practice connects the ontology of our study (our ways of being together) with the epistemology of our study (how knowing shapes experiential knowledge and coproduced knowledge) and the doing (the practices for how we work together). Ideally, these ways of being, knowing, and doing shape ways of seeing through the implementation of the ideal relation of being-for (explained next). In Table 1, the component parts of the Co-Design Living Labs program, within which the philosophy of practice is operationalised, are detailed.

**Table 1:** The component parts of the co-design living labs program and future network.

The component parts of the co-design living labs program and future network	How the component parts are implemented and operationalised
Membership	Invitations are sent via completed mental health research projects. We ask for information such as name, age, gender, contact details, lived-experience context, preferred modes of contact. When people join the program and network we refer to them as co-designers rather than members to develop relational ways of working. Co-designers are from different backgrounds and across the life course.
Registry/database	A registry/database is maintained by a trained data manager using Redcap. The database is used to track who is invited to co-design and who attended co-design annually. It is used to share invitations to co-design or priority setting or other co-design activities. In our registry we have more details that enable us to match co-designers with topics and we can identify when people may not have engaged for a while.
Onboarding/orientation	New co-designers receive a link to the co-created Living Handbook. This is ‘A handbook by and for working with co-designers’ The examples of co-design within the handbook are also updated to stay current. People are sent an introductory pack and encouraged to introduce themselves as new members in a co-design session.
A Living Co-Designer’s Handbook	The living handbook is called such because it is added to by new co-design co-leads and people as new information is needed. This includes the history and overview of the Co-Design Living Labs program and now national network. The handbook includes preparatory and post co-design self care tips from other co-designers. There are preparatory questions that have been established for people to complete ahead of attending co-design which ask for information about gender, preferred communication modes, and recieveing information. An Aboriginal and Torres Strait Islander version of preparatory questions is also included and our working together agreements (also called Principles of Participation) are included in long written form.
Continuous engagement/relational engagement	Co-designers receive support from our Co-Design Living Labs coordinator and network research lead to attend sessions through taxi vouchers when attending in-person or zoom support/instructions for online co-design. Supportive phone calls can be made for further explanation of what to expect in co-design. If we have not heard from people for a while we phone to invite to co-design and to re-establish connection. Regular updates are shared by co-leads of the program and network every second month. An annual newsletter is sent to all members electronically and in hard copy. An Open House drop-in was established more recently in 2023 to facilitate connections across membership and co-lead groups.
Convening co-design virtual and in-person	We operationalise our philosophy of practice ‘Togetherness by Design’ through using our working together agreements, ensuring boundaries are set and members feel safe enough. Only selected convenors and consistent core team group convene (rather than people coming in and out of convening who are unfamiliar). People with shared lived-experience are the ideal convenors but where this is not possible we co-partner in the approaches. Co-design convenors used narrative methods, participatory approaches and are informed by different design approaches and design thinking techniques. A majority of co-design is conducted virtually enabling wider participation across the nation. We use a digital whiteboard to facilitate online co-design.
Co-learning and knowledge transfer strategies	All outputs from co-design are shared back to co-designers for further feedback. If a project is completed, co-designers receive an update on this and a link to the new Co-Design Living Labs network pages online for sharing stories to encourage co-learning and knowledge transfer across settings.
Respect for time contributions	Co-designers are always reimbursed following our paid participation policies within the ALIVE National Centre for Mental Health Research Translation. We will always reimburse for co-design meetings (called sessions) and co-researchers are appointed as paid casual researchers, or as research fellows and associates within the university system.
Co-leadership model	The national network is led by co-designers who form co-lead groups across the 17 university partner nodes. Capacity building is provided through co-lead roles and mentorship is supported by existing co-design research staff within the team. Capacity is being developed further through a specific Co-Design Trainee Award program to foster leadership from within co-designers.
Evaluation/Expanding our explanatory theoretical model of change for co-design and co-production	Continuous feedback after each session on co-design facilitation and processes. We use our established explanatory theoretical model of change for co-design and co-production to evaluate co-design process and outcome. The eight mechanisms of change identified within the theoretical model help guide the processes used within co-design (as articulated in the working together agreements) and the whole theoretical model of change is applied for evaluation.

**Figure 2:**
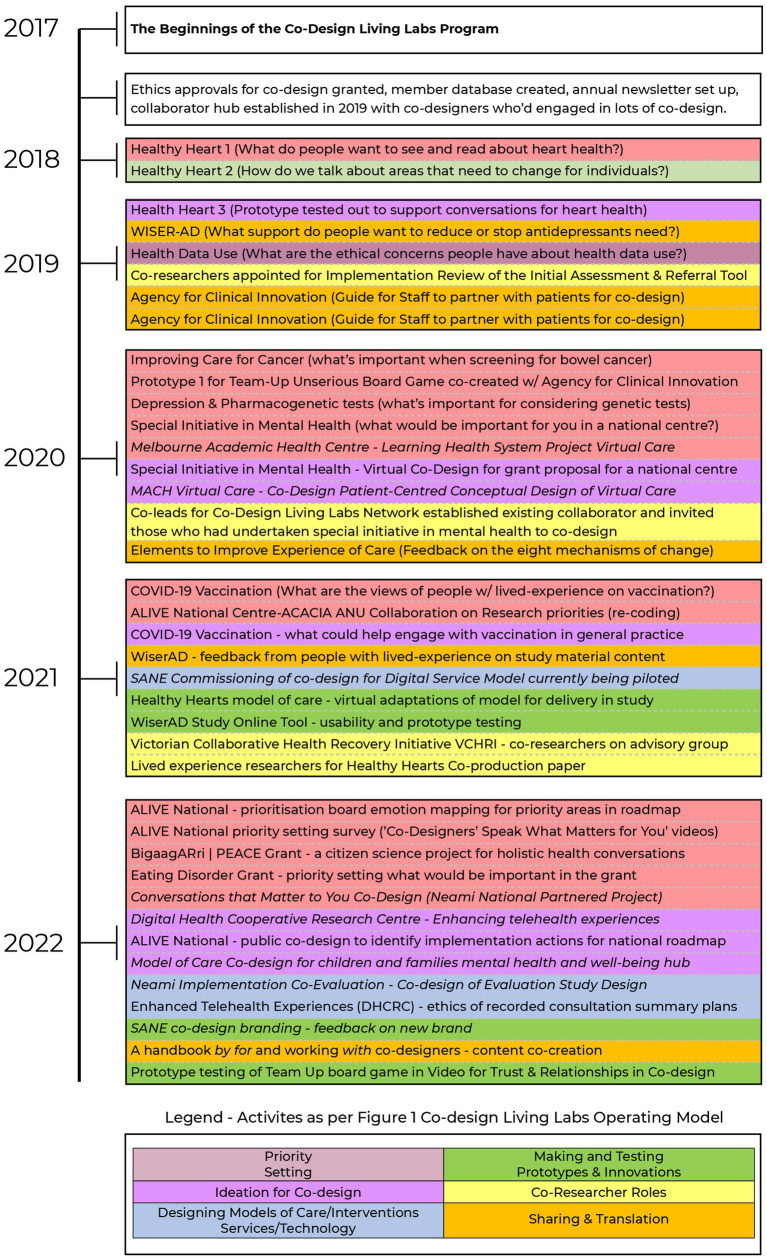
The evolution of end to end research design to translation activities in the co-design living labs program (2017-2022).

We describe the evolution of the program with reference to selected examples of co-design activities undertaken during 2017–2022; the full overview of co-design conducted between 2017 and 2022 is presented in Figure 2 also.

## 3 Results—development of a philosophy of practice “Togetherness-by-Design”

### 3.1 Ontology—ways of being

As noted earlier, the implementation of our philosophy of practice hinges on a specific commitment to ways of working and being together that draws on three forms of togetherness. These forms of togetherness were originally outlined by Zygmunt Bauman and drew heavily on the work of philosopher Emmanuel Levinas (22). Bauman described the forms as being-aside, being-with, and being-for. These are types of relations exist in our everyday worlds and can be practiced (and not practiced) between people. The three forms of togetherness provide the program with a guide to how we work collaboratively with co-designers and partners of our program.

To understand what is meant by the being-aside relation, it is helpful to think of a physical space that is shared between two parties or entities (beings). In this space, these entities may indeed be co-present, but there is no recognition by any of the entities that the ‘other’ is there, has any importance, or is even “person-like”. This relation can be seen, for example, when people get on and off public transport. There is shared space but no recognition of each other; we move aside and move on. Being-aside offers a way to understand inhumane engagements characterised by people occupying physical spaces aside from each other but not seeing or acknowledging the person, the identity, or experience (30). There is no sense of need for this recognition nor associated connection in being-aside relations. It leaves people feeling deeply isolated and disconnected from each other; unseen, unheard, and unknown. For this reason, being-aside is an important relation to be aware of in ways of working in co-design—it might even be said to be the antithesis of co-designing together.

In contrast, being-with relations advance beyond the reality of occupying space together towards some recognition that there are others around us. Unfortunately, in being-with relations, this recognition is based largely on interests. Being-with, according to Bauman and his use of Levinas’ notion of the Other, is an encounter of ‘no more than the topic at hand permits’, and once exchanges have been made, nothing more evolves or continues; no more of the self is given to the encounter than the transaction that underpins it. At a community level, being-with is played out at the shopping centre with short hellos, exchanges of money for products, and a departure from the setting without further thought given to the encounter or those within it.

The ideal form of togetherness, according to Bauman (22), is being-for, where people and beings are honoured as contributors to relationships regardless of the status they bring. In being-for, actions are always oriented towards a dialogical connection—that is, my story is connected with your story, but it is not my story to share, it is always incomplete, and I can never close this off. This is a relation we share in and should be seen as beyond individual one-to-one notions of engagement and expanded to communal worldviews. In Levinas’ conception of the Other, it is a totality that can never be entirely and fully known, but it is a relational connection that persists beyond time and space. For Bauman (drawing on Levinas), one must *be for* the Other before one can *be with* the Other (31). This means seeing the face of the Other and coming to share responsibility with each other.

As a philosophy of practice, togetherness by design enables the Co-Design Living Labs program activities to move from the transactional space of being-with to enact being-for as the relational goal from which we work together with co-designers and communities beyond the university. This contrasts with the way communities have traditionally been invited to participate in research, which has largely been more reminiscent of Bauman’s concept of being-with. Sadly, in some instances, being-aside has also characterised research endeavours where there has been active exclusion, avoidance, and lack of engagement with some communities. Togetherness-by-Design, with its orientation to being-for, also enables us to share unconditional responsibilities for and with each other.

**Figure 3:**
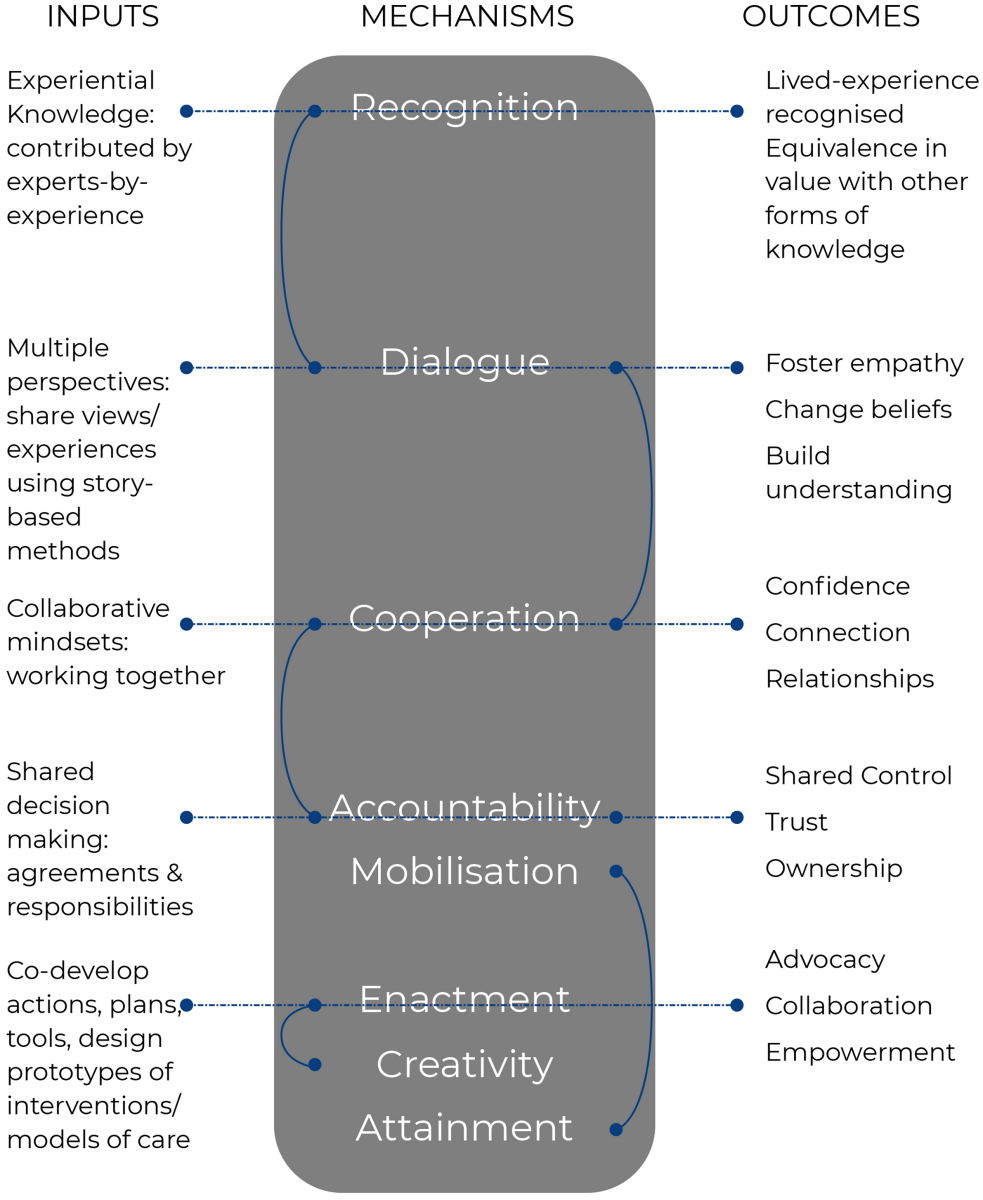
Adapted mechanisms of change as applied within the Co-Design Living Labs program.

To create the conditions for change and a commitment to being-for, eight mechanisms of change are employed to set relations and evaluate Togetherness-by-Design. These mechanisms are presented in Figure 3 and have been adapted from an existing explanatory theoretical model of change for co-design and coproduction in healthcare improvement (1). The mechanisms have informed our working together agreements that we articulate in each co-design session for creating safety and shared understanding for co-design. Coupled with the theoretical model, these mechanisms can also be used to evaluate co-design processes and outcomes.

The Co-Design Living Labs philosophy of practice thus expands university–community relationships beyond transactional one-off research engagements where they are based on no more than the topic at hand permits (25). Evaluative evidence from 11 of the co-designers, who contributed to the development of the conceptual design for a digital service model for people with complex mental health needs (32), suggested that the practice of ‘Togetherness-by-Design’ does foster connections and dialogical relations consistent with the goals of being-for. The co-designers comprised both existing members within the program and people external to the program who were invited from partnered organisations also. The co-designers, when they were asked about the sessions, said: ‘people felt that the group members did not talk over each other, people did not have to compete to feel heard, everybody had a chance to talk, the group was accommodating of a diversity of experiences, the ways of working were open, respectful, and the activities using pictorial descriptions to talk about experiences and ideals were valued’. Co-designers who provided this feedback also added that, for them, ‘feelings of empowerment were fostered by their voices being heard, the engagement was enjoyable and met their hopes for coming to co-design’. The hopes people said that they had for engaging in the co-design included ‘the importance of being a part of solutions, creating positive change, creating better services and sharing experiences, and that a new set of ideas that others may not have thought of may emerge’. Importantly, the feedback included aspects of discomfort and challenges that co-designers had observed. Some co-designers sensed anger within the group, but the comments on this indicated that they remained comfortable expressing their discomfort around this without needing to close-off the anger or other person’s experiences. This illustrates the operationalisation of being-for as part of our togetherness as, despite conflict, the group managed to still see each other in all the forms of human expression and be in that together.

### 3.2 Epistemology—how knowing shapes experiential knowledge and coproduced knowledge

Togetherness-by-Design is further enacted by applying being-for to ways of knowing. Here, lived-experience is seen as a form of knowing that is essential to what is coproduced. The Co-Design Living Labs program has grown from following experience-based co-design in the early establishment phases to employing Togetherness-by-Design in its practices and processes. Originally established by Bate and Robert for service and quality improvement (33, 34), experience-based co-design (EBCD) commonly refers to two stages in a service or quality improvement process. Stage one is information gathering and stage two is co-design; these two stages are interconnected and integral to each other, so they should not be seen in isolation. What is important to highlight here is that the methods used to understand and elicit experience within experience-based co-design are deeply centred around narrative, participatory methods, and learning theory. Narrative is key as it centres on how socio-cultural contexts matter in identities and as a method; it values identity as central to the experience. Experience-based co-design enabled the program to enact being-for as a relation instead of falling to other methods where being-with might be the norm. An example of this might be in research activities where we ask for no more than the topic at hand permits. For example, structured surveys might be a case in point where often, when views have been exchanged in a question-and-answer format and submitted, the interaction is complete. The engagement is momentary and passes; nothing more occurs beyond this transaction, and often further interaction and engagement are discouraged in the style of survey administration. With experience-based co-design and its emphasis on narrative, we have fostered dialogical approaches to connect with peoples’ stories, identities, and values.

As indicated above, the Co-Design Living Labs program has therefore been shaped by narrative, learning theory, participatory, creative arts, and visual methods to elicit and shape experiential knowledge for co-design. This has ensured that experiential knowledge is key to what is made and shaped. The central focus on coproduced knowledge reflects the enactment of being-for as our relational way of working. The program also importantly builds on the living labs tradition of open innovation, collaboration, and partnerships across community, industry, and government to achieve this ideal (35, 36).

In the Co-Design Living Labs program, experiences, therefore, foreground and shape all activities, and our goal is for an epistemology that elevates experiential knowledge so that it is afforded what Fricker termed “epistemic justice” (37). In this respect, we are interested in how justice in the context of knowledge can be both discriminatory (how experience is valued, heard, and acted on, for example) and distributive (how goods are distributed, for example, through information sharing or education) (37). Justice also operates within the knowledge production processes of research and the institutional settings where it is carried out. Therefore, embedding co-designers within the leadership of the program and the now national network has been important as a strategy for the distribution of justice.

Our study indicates that experience is a fundamental first premise, and this is noted within the eight mechanisms of change (as presented in Figure 3) as essential for shifting to novel interventions and models of care that can facilitate lasting change. This means the experience drives not only what is improved in healthcare or other settings but also extends to what is researched, the research questions, and the research process from the study design to the translation processes; experience is embedded within the fabric of the program of study. This is shown in the Figure 1 model of operation. Although, critical measures of success must be about more than togetherness. They must necessarily move towards evaluating if the implementation of co-designed research projects and models of care is effective. The questions must be asked as to whether co-design results in structural and systemic shifts in power, addressing social injustices and ultimately creating better healthcare experiences and outcomes.

The Co-Design Living Labs program mirrors many elements of the arrival of the era of coproduced knowledge (38). This era of coproduced knowledge is seen as a push towards the valuing of experiential knowledge equally to that which is generated through medical and scientific positivist-focused research. Expertise based on experience has often been neglected due to the associations of subjectivity, the view that individual perspectives may be potentially biased and too subjective, and the complexity of experiential knowledge (39). Sociologist Borkman (40) referred to “experiential knowledge” in the 1970s as “the truth based on personal experience with a phenomenon.” Experiential knowledge is described as holistic and emerge from the multi-faceted and ongoing experiences of living with a particular condition or experience (41). Our philosophy of practice acknowledges that different forms of togetherness bring multiple ways of knowing; what is key is to share in the understanding of these for future change. This means honouring community-led lived-experience and ensuring this knowledge is at the heart of co-design practices.

Our enactment of being-for to coproduce knowledge and new systems for implementation research was most recently demonstrated in the vision for the ALIVE National Centre for Mental Health Research Translation, for which a short case story can be read here.^1^ Following the funding scheme being announced for the ALIVE National Centre, we sought to establish what the research priorities for our co-designers might be to ensure the grant proposal reflected the priorities of people with lived-experience of mental ill-health and carer/family kinship groups. To do this, three open-ended questions were circulated to co-designers by email as follows:

1. What would be important to you in a national research centre dedicated to mental health research?
2. What are the main areas you think researchers should be looking at in mental health? What are the vital signs that we should be doing better in within mental health?
3. How would you like to be involved in a national centre, for example, would you participate in training activities for research, workshops about mental health research, meetings to network and grow expertise, or would you want to be trained to be a researcher?

**Figure 4:**
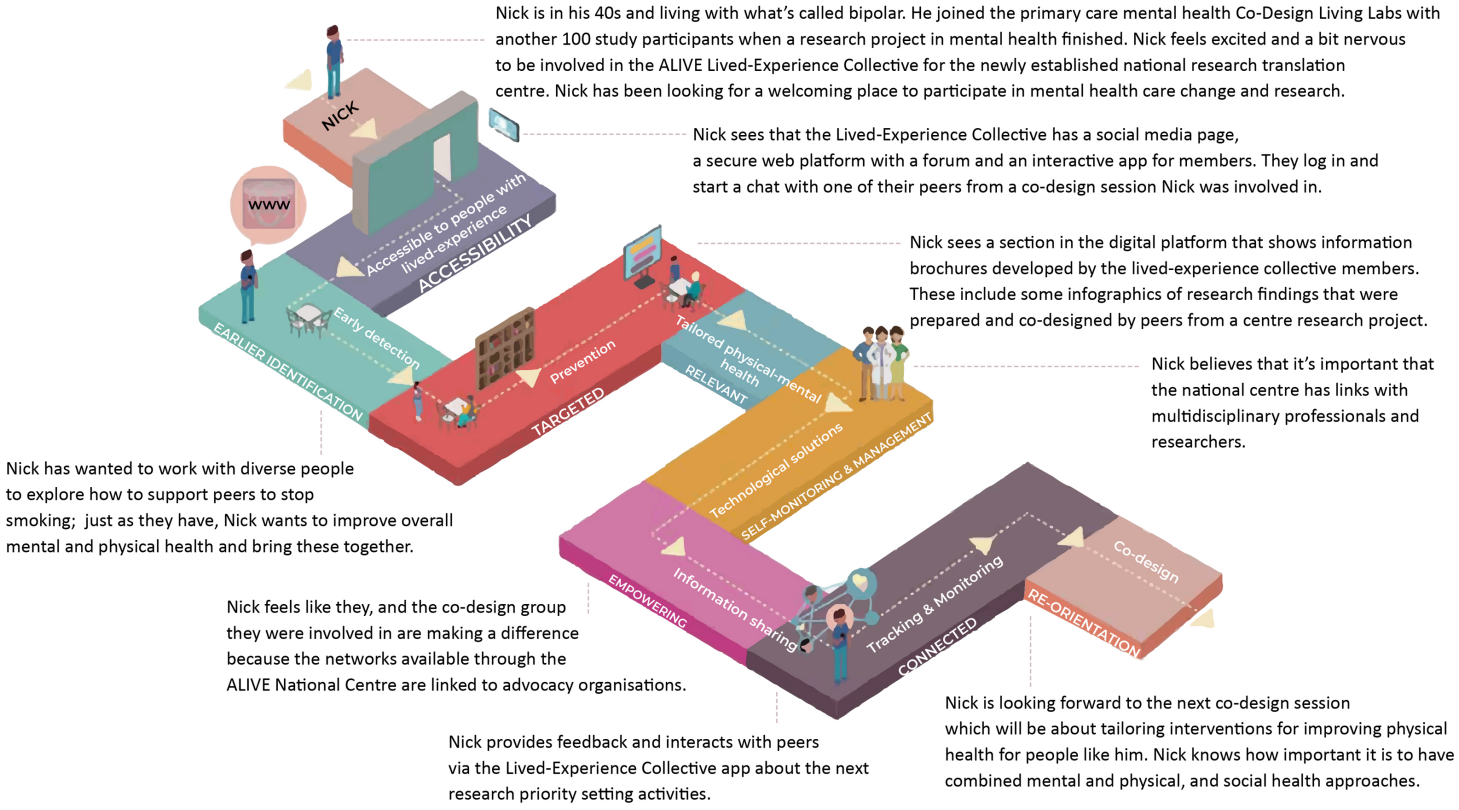
Conceptual design of the ALIVE National Centre for Mental Health Research Translation research priorities and experiential journeys.

The email request was circulated for 2 weeks, and priorities were read by the lead for the Centre proposal (Palmer) and two researchers within the Primary Care Mental Health Research program. Responses were organised into thematic statements to formulate the research programs and their objectives. The overall experiential journey that someone with lived-experience of mental ill-health might have in the ALIVE National Centre was co-designed with 27 co-designers in three further ideation sessions. Of this group, eight co-designers were then named as co-leads within the grant proposal. Figure 4 presents how the thematic statements and priorities for research were translated into an ideal journey of a co-designer called Nick for the Centre’s proposal.

The pathway Nick takes shows the research priorities as the foundational blocks of the path and the experiential elements that were ideated on within co-design meetings along the top of the path. These experiential elements for the ALIVE National Centre included accessibility of services, early detection, prevention, tailored physical and mental health responses, technological solutions, for information sharing, tracking, and monitoring, and co-design. The research priorities (foundational blocks) were articulated similarly for the goals of delivering at-scale mental healthcare. The goals included accessibility, earlier intervention, prevention, targeted, relevant, self-monitoring and self-management, empowering, connected, and reorientation. Importantly, this is an assemblage of a range of people’s priorities and views constructed for grant purposes, and we note that experiences rarely ever mirror a neat linear path. The desired journeys articulated by people with lived-experience through the ALIVE National Centre are shared in the text as an example. This ideation and shaping of a Centre vision reflect coproduced knowledge in action whereby people with lived-experience have set out priorities and the experiential journey within the Centre, and this has subsequently been implemented within the Centre’s establishment and operationalisation.

### 3.3 Practice—ways of doing and undoing co-design

Ways of being and knowing that privilege being-for are critical to how we practice co-design in the program. We recognise that some existing tools and techniques for co-design need to be evaluated for whether they support these ideal relations (42). Currently, the processes and techniques within the Co-Design Living Labs program draw on participatory design practices, narrative approaches, participatory action research, visual methods, and creative arts-based methods (43). However, this still means that design methods need to be continuously re-evaluated and appropriate cultural framings applied. There must be a critical evaluation and expansion of the kind of co-design techniques and processes used. It is important to also implement ways of undoing co-design. For example, we critically evaluate whether personas are essential to the co-design processes given that there are risks of these being unintentionally stigmatising with stereotypical representations. To ensure cultural security and intersectionality is respected within co-design, all methods require rigorous evaluation and consideration of appropriateness for identities and existing knowledge systems—being-for the other precedes working together.

Implementation of Togetherness-by-Design has seen the program shift its language use from Co-Design Living Labs program members to ‘co-designers’, as explained in the introduction. This is an important signification of collaborative relationships, where power can ultimately be shared rather than represented as a member–researcher relationship, signalling a transactional value system. Researchers within the program have had to unlearn and shift beyond the terminology of research subjects or participants and see people as they are, as individuals who bring their life stories and experiences to support research design and translation activities. This has been an important step in moving from researcher-initiated processes to increasingly co-designer-led approaches. In valuing experiential knowledge and contributions, we therefore seek to recognise this in authorship practices and, in the longer term, in moving to a community-owned (social enterprise) model. The recognition of contributions and co-creation currently varies from the co-created pieces generated. Many co-designers prefer the use of first names only in some outputs to retain privacy or protect safety where they may be survivors of abuse, intimate partner and/or family violence. Some co-designers have become co-researchers within research teams and others also contribute actively to paper writing, editing and crafting work. Others are named on research grants as co-investigators and not solely as advisory group or committee members or as associate investigators which can be a dominant research practice. Co-designers are always reimbursed for their time contributions to co-design sessions—illustrating the importance of resourcing within the architecture and the component parts of the program (shown in Table 1). The role of the facilitators (or conveners) is to support engagement in co-development, to provide explicit frameworks, to share decisions and facilitate power-sharing arrangements, and to co-design and then use this to synthesise and develop either a set of design principles or a tangible artefact or model of care as required. At a deeper level, operating in a being-for relation also means changing our relational ways of connecting inwardly and outwardly for change to be sustained.

Co-design sessions have largely used whiteboards (digital whiteboards when virtual—a lot of co-design has been online since the COVID-19 pandemic onset in 2020). Experience and journey maps have been co-created and explored through facilitated group discussion, and final outputs were created by using emotion mapping within processes. The activities used within co-design sessions are usually selected to be matched with the co-design objectives and the experiences of people in the room; it is important to reiterate that our ways of doing are constantly in motion and changing. These reflect the evolutionary trajectory of the program over time. In current practices, many linear maps have been replaced with circular models reflecting how people with lived-experience engage in story-telling and sharing their experiences. Table 2 presents some greater detail of the adapted methods that were used within co-design sessions of the new digital service model for SANE Australia that the ALIVE National Centre for Mental Health Research Translation conducted with an explanation of why these methods were used. These methods are overviewed in multiple articles but Milton and Rogers’ research methods in product design succinctly describes them (43). The table illustrates how these were adapted within our co-design approach.

**Table 2:** Ways of practice – examples of design activities used in the co-design of a conceptual model of care for a digital service model for people with complex mental health needs.

Aim	Co-design method/s	Purpose/application
Understand current experiences in the service system and future ideal. To surface negative feelings and experiences early for setting out foundations to work together. To create a sense of a future space to work toward. Understand a technology (program, app, website) or general use of digital health in people’s everyday life.	*Photo Artefacts* – Here, people are asked to provide ahead or bring to the start of a session, an image or to take a photo in their local community that they feel could help to describe the current mental health system. People are also asked to include a photo of the future ideal system. *A Day in the Life –* Mapping exercise used within co-design session to identify where, when and how (if at all) technology or digital health broadly may be used in day to day life. Outlines a clock and provides time for anchoring when usage occurs and for what reason people are using the technology.	This activity is set out ahead of a co-design session. It is ideal because it does not take people long and they can bring an existing image from home or take one for the co-design. Helps to generate an understanding of service system views from individuals and across the group. Helps to surface negative views and possible experiences within a system to inform what not to re-design or current sticking points. Asking for an ideal photo with current system scaffolds activity with a sense of future change. Helps for introducing self and others as focus is on image and not the person too. Orientation toward the kind of technologies used and within the day to day of people’s lives, indicates the areas where there is good and not so good fit. Helps to create a picture of enablers and challenges for the new service model or technology and what kind of ways people might engage with this – and what kind of needs people might have to be supported to use technology or digital health further.
Eliciting the desired experiential goals/values of models of care or new service models or an intervention and new product.	*Emotion Maps—* Use a journey board/map to capture experiences and the touch points through service journeys. The positive and negative experiences are shared through the journey map and this enables people to share strong and not so strong feelings about these experiences. The process can result in identification of consensus within groups on the negative experiences which indicates the areas for change.	To elicit the service journey touch points (the places people come in touch with different parts of the service system/organisation or topic, which shape experience positively or negatively) – illuminates values and experiences of people and leads to the identification of the experiential goals of people with lived-experience for conceptual designs.
Identifying and co-designing the services that people would like to access in a model of care or new service.	*Menu of Services—* Placing ideas of services into a menu format and using the entrée, main, and dessert to organise service features from entry to the service through to follow up. This is a beneficial approach because it creates themes of nourishment, working together collectively, and all being around the table to share in service identification.	Identification of service elements that people are seeking and concepts of importance. The type of groups, the accompanying services, planning support and features all enable the development of the foundations of conceptual design. The concept designs usually draw additional material together from other activities undertaken within co-design—for example, they bring together emotion map desired experiential values and goals and service concepts to be visually represented together.
Identification of choices between core service concepts to inform the service design and development. Expanded model of care for implementation	*Card sorting—* Prioritising services that can be included or features of services. Working together to think about the component parts of the core service concepts and what choices may need to be made. *Film review—* Short film roll approach showing the journey exploration through different representations of people’s goals, desires and experiences shared. A digital story is also co-created here to share the journey together.	Formulating further the preliminary design concepts and principles of the service model and understanding what is most important to users. Enables shortlisting if needed and consensus based conversations. Sharing the service concepts and component parts with different groups who will be responsible for implementation to ensure that the experiential goals are shared and to identify divergent points of view. Digital story for further co-design and a focus on what strategies might be needed to support implementation into practice, system and services. Digital stories help to remind people of the co-design journey that has been undertaken and the core touchpoints that were identified including providing evidence that experiences are shaping the new model or technology, and voices being incorporated alongside providing potential for celebration.

The commitment to co-creation and genuine/equal collaboration within the Co-Design Living Labs program extends to the data analysis stages of research and co-designed outputs, as well representing our being-for commitment to epistemic justice. To facilitate this, conceptual designs are shared with co-designers and expanded upon before delivery to partners. This is in keeping with fostering shared decision-making throughout co-design processes so that people are making choices about how input is configured and shared; this, in turn, embeds lived-experience at the heart of the conceptual designs and outputs. The Co-Design Living Labs program has ongoing ethics approval from the university human research ethics committee, which enables co-design to be responsive and iterative. This provides us with the capacity to run a continuous model of co-design to service the Australian mental health sector in the future and to embed lived-experience within research and improvement, re-design, and change efforts in implementation and translational research. Since we commenced in 2017, we have evolved working together agreements from our eight mechanisms of action (as noted in a previous section, and referred to as well as principles of participation), which are shared at the commencement of each co-design session (8). The eight mechanisms are part of an explanatory theoretical model of change that enables our program to enact continuous learning and evaluation of activities as well. Figure 3 details these adapted mechanisms within the program.

### 3.4 Ways of seeing—continuous learning and expansion of the program

The theoretical explanatory model of change enables documentation of experiential knowledge as a central driver in changing mental healthcare systems and to appreciate the concept of recognition as critical to change (44). It is important to also note that while the eight mechanisms appear neatly listed, they are not hierarchical and always remain interconnected—the intent is not for a usual program logic pathway that follows a, if this, then that, will result. In this respect, recognition and dialogue are critical to the shared understanding of narrative and storying, and the acknowledgement of polyphony (many voices) within co-design. Cooperation is then enacted through the sense of solidarity for communal causes, working together, and developing a shared agenda for making change. Accountability enables the shared agenda for change to grow through motivation as a group and agreeing to mobilise to make change happen. In this model, the co-development of actions must be accompanied by enactment of these actions with creative implementation to attain change. Actions without implementation lead only to more co-designed ideas without lasting change.

## 4 Discussion

The establishment of a Co-Design Living Labs program and the evolution of its philosophy of practice have been described in this paper. Using examples of co-design that have been undertaken, we have outlined the philosophy of practice for the program, Togetherness-by-Design. The key purpose of the Co-Design Living Labs program is to ensure that lived-experience is at the heart of mental health research, service design, delivery, improvement and evaluation, and research translation. Having recently celebrated its fifth year of operation, this article has shared the retrospective story of the evolution of the program. It has illustrated how Togetherness-by-Design is enacted across the model of operation by a commitment to being-for as an ideal relational ethic that shapes the ways of being, knowing, and doing in our work. The program architecture has resulted in component parts of the program that are fundamental to the realisation of our vision. These component parts have included a research-managed registry/database since inception, which has facilitated continuous replenishment of the membership base and coordination of activities in a structured approach. Having a coordinator to invite and support people to come to co-design, whether in person or virtual, has been a critical ingredient for togetherness. This is possibly because co-designers feel connected with and in a relationship with the research team. In addition, the registry/database has enabled focused engagement efforts as it is possible to see who might not have engaged and connect with people via telephone to hear more about what people would like to be engaged in.

As a successfully funded research program, there has been continued opportunity to grow the community base of co-designers, as reflected in the recent establishment of the Alex McLeod Co-Design Training Award in 2022. This award supports two co-designers per year (until 2022–2026) to hold paid part-time roles to learn about program operations and to grow co-design capabilities for future leadership. Our growth of a registry/database living labs approach ensures that much diversity can be reflected within end-to-end co-design. In the future, we will become a program that has membership across the life course and expands with an Aboriginal and Torres Strait Islander-led component of study. Saying this, it is important to acknowledge that the Aboriginal-led program may have cultural practices and approaches that connect with, but are different from the current approaches. Additionally, there are always gaps and limitations within research structures and impositions on the conduct of activities that are funded. For example, research projects where co-designers have been recruited to date may have had specific eligibility criteria, which means that certain groups have been excluded due to the language requirements of those studies; thus, we must ask how to improve these situations. Ensuring that there is space for togetherness in these circumstances may require more attention as we move into the future. The program may not reach people who do not want to engage within what might be perceived as traditional research structures or who seek to represent their experiences in different ways to what is on offer. This is where co-partnership and relationships beyond the Co-Design Living Labs are key. As the Co-Design Living Labs network expands nationally, the membership base will necessarily need to reflect greater diversity for different local contexts. Currently, where gaps exist in the program, when we undertake co-design, we ensure that we co-partner with organisations to embed community-led approaches.

The Co-Design Living Labs engagements (or sessions) are typically short-term and entirely opt-in. We are conscious that there is potential to create high demand on people with lived-experience and carer/family and kinship group members around requests for co-design, and there are always risks of programs such as this being seen as service models—things that exist to serve other organisational needs. There will be a need to turn attention outward to explore how being-for is maintained as the ideal relation in these instances of collaboration. Given that government relationships are largely transitory and often replicate being-with as the primary relation, the opportunities for transformation from co-design will be limited. The registry/database provides the potential for the research team to manage invitations and for targeted in-reach to co-designers with specific expertise. The program also shows how it is possible to ensure community-led lived-experience is at the forefront of mental health research and that it can be embedded within traditional academic structures and work towards power-sharing despite known hierarchical systems. Saying this, however, it must be acknowledged that human resource systems are not well-designed in university contexts to support reimbursement of co-design activities and funding to ensure that participation is paid appropriately can be a challenge. When co-designers move into co-researcher roles, the pathways for engaging in research and career development are also poorly designed. The establishment of the Co-Design Living Labs program reflects how universities ought to aspire to engage not only *with* but with the relation of *being-for* communities. Being guided by Bauman’s (22) three forms of togetherness, the philosophy of practice Togetherness-by-Design supports a reorientation of research hierarchies in the process of working together and disrupts power-laden practices of *who* decides what is researchable and *how* this is undertaken. As the model expands and disrupts its own traditional structures with Aboriginal and Torres Strait Islander community-led research in focus in the ALIVE National Centre for Mental Health Research Translation, being-for will be a critically important foundation to re-distribute power for community-led approaches (23). Ways of doing may simply need to become ways of undoing in some circumstances.

As all funding agencies increasingly implement the prerequisite for co-design with people with lived-experience of mental ill-health and carers/family/kinship group members within grant proposals and research (18), and as health researchers increasingly dabble in co-designed interventions and digital health technologies, it is critical that the processes and methods used for co-design are better articulated, understood, and evaluated. One example of improving the information shared was recently provided by Knowles et al. (45) in a description of Public Patient Involvement (PPI) within a United Kingdom (UK) Learning Health System (LHS) project. In that article, the co-design questions, method, and proposed outcome were detailed within an overarching table to show rationales and intended outcomes; we have emulated this within our study in Table 2 to follow good practice. This is a positive first-step practice for health researchers who undertake co-design to include as part of the processes of describing interventions, model of care, or technology development. However, we must also be conscious of the need to couple this with detailed overviews of ways of working that articulate clear philosophies of practice so that the relational focus of co-design is not eroded, overlooked, and subsumed. Additionally, evaluative frameworks for the impacts and outcomes of co-design are required—this includes paying greater attention to whether structural and systemic injustices are remedied from co-designed research and how impact and outcomes might be measured. It also means acknowledging where change within established co-design programs of work might be needed in keeping with the dynamic, changing, and shifting nature of co-design more broadly. One theoretical model of change designed for evaluation has been presented within this article as useful to setting conditions for co-design, understanding processes, and evaluating for impact at individual, social, community, and organisational levels (1).

## 5 Conclusion

As the Co-Design Living Labs program moves from a local program model to part of a national network with the ALIVE National Centre for Mental Health Research Translation, fostering capabilities within local university nodes will be important. The key characteristics of the approach, the dedication to relationship formation, and the commitment to Togetherness-by-Design as the philosophy of practice must remain front and centre. Feedback from co-designers has suggested that Togetherness-by-Design has supported the goals, values, and processes of co-design processes and outcomes. This indicates that Togetherness-by-Design helps to realise the mechanisms of change (recognition, dialogue, cooperation, accountability, mobilisation, enactment, creativity, and attainment) in co-design. This means shared values that facilitate relational ways of being, knowing, and doing and a full appreciation of the distinctions between non-relational and transactional ways of working (being-aside and being-with) with relational ways of togetherness (being-for) are even more important. The Co-Design Living Labs program represents one example of an adaptive and embedded approach for people with lived-experience of mental ill-health to drive mental health research design to translation, which can be delivered at scale. These approaches need to be embedded in architecture across research, government policy and practice, and service settings. As scaling commences, the emphasis on co-leadership from co-designers and the transition to a living labs cooperative social enterprise model will become key.

## Data availability statement

The original contributions presented in the study are included in the article/supplementary material, further inquiries can be directed to the corresponding author.

## Ethics statement

The studies involving humans were approved by The University of Melbourne Human Research Ethics Committee. The studies were conducted in accordance with the local legislation and institutional requirements. The participants provided their written informed consent to participate in this study.

## Author contributions

VP conceptualised the establishment of the Co-Design Living Labs program using participatory design expertise and knowledge from the world first trial of adapted co-design for mental health settings they led and developed the philosophy of practice with input from co-designers over the life of the program. JB co-convenes co-design activities and leads research activities within the program and supports co-lead group meetings. ML has co-convened healthy hearts project related co-design sessions with VP and works regularly with ED who is a lived-experience co-researcher from the program. KD established the registry/database for the program using redcap and provides statistical updates on membership, participation and withdrawals. RK coordinates invitations to join the program and sessions within the program and supports KD for analyses. ED, PS, AD, BH, TS, ND, and GM are co-leads within the program (with an unnamed carer co-lead) who meet once a month to guide progression and activities of the national network and foster leadership within the program. All authors contributed to the article and approved the submitted version.

## Footnotes

^1^https://alivenetwork.com.au/our-networks/co-design-living-labs-network/case-study/case-study-2/
